# Extraction of Polysaccharide from* Spirulina* and Evaluation of Its Activities

**DOI:** 10.1155/2018/3425615

**Published:** 2018-04-11

**Authors:** Bingyue Wang, Qian Liu, Yinghong Huang, Yueling Yuan, Qianqian Ma, Manling Du, Tiange Cai, Yu Cai

**Affiliations:** ^1^College of Pharmacy, Jinan University, Guangzhou 510632, China; ^2^Guangzhou Jiayuan Pharmaceutical Technology Co., Ltd., Guangzhou 510632, China; ^3^Guangzhou Guoyu Pharmaceutical Technology Co., Ltd., Guangzhou 510632, China; ^4^College of Life Sciences, Liaoning University, Shenyang 110000, China; ^5^Cancer Institute of Jinan University, Guangzhou 510632, China

## Abstract

**Background:**

Polysaccharide of* Spirulina platensis* (PSP) is a kind of water-soluble polysaccharide extracted from* Spirulina platensis*. It has been proved to have antitumor, antioxidation, antiaging, and antivirus properties. And it has a promising prospect for wide application.

**Objective:**

This study aims to identify an extraction process for high-purity polysaccharide in* Spirulina* (PSP) through a series of optimization methods and then evaluates its initial antiaging activities.

**Methods:**

Four kinds of extraction methods—hot-water extraction, alkali extraction, ultrasonic-assisted extraction, and freeze-thaw extraction—were compared to find the optimal one, which was further optimized by response surface methodology. PSP was obtained after the crude PSP was deproteinized and depigmented. The antiaging effects of PSP were preliminarily evaluated through in vitro cell experiments.

**Results:**

The alkali extraction method was determined as the optimal method, with the optimized extraction process consisting of a solid-liquid ratio of 1 : 50, a pH value of 10.25, a temperature of 89.24°C, and a time of 9.99 h. The final PSP contained 71.65% of polysaccharide and 8.54% of protein. At a concentration of 50 *μ*g/mL, PSP exerted a significant promoting effect on the proliferation and traumatic fusion of human immortalized epidermal cells HaCaT.

**Conclusion:**

An extraction method for high-purity PSP with a high extraction rate was established, and in vitro results suggest antioxidation and antiaging activities.

## 1. Introduction

*Spirulina* is a genus of edible and nutritious prokaryotes, which contains up to 60%–70% protein, 6%–12% polysaccharide, and a variety of vitamins, fatty acids, and minerals [1]. Moreover,* Spirulina *exhibits biological activities in the human body and plays a unique role in the fields of medicine and healthcare [2–4]. Polysaccharide in* Spirulina* (PSP) has been reported to have antitumor [5, 6], antioxidation [7, 8], antiaging [9], immune regulation [10], and antivirus activities [11, 12]. In addition, PSP has effects on anti-inflammatory [13], antiradiation, antifatigue, and antimutation activities [14–16]. The activities of polysaccharide vary with the structure and existing form. Thus, the structure needs to be kept intact during the extraction process.. A wall-breaking method, such as ultrasound and microwave, is required before extracting plant polysaccharide [17, 18]. Common extraction methods include water extraction [6, 19], alcohol precipitation, acid-base extraction, ultrasonic extraction [20], freeze-thaw, and enzymatic hydrolysis [21].

## 2. Materials

### 2.1. Main Experimental Equipment

The experimental setup included High Performance Liquid Chromatography (Agilent 1260, RI-II), a pH meter (pH 10/100, Shanghai Di Yim Instrument Limited Company), an analytical balance (Q/SGYM1008, Austrian House Equipment Co., Ltd.), a decolorization shaker (TS-1000, Haimen Bell Instrument Manufacturing Co., Ltd.), and an inverted fluorescence microscope (TS100, Nikon Company).

### 2.2. Main Experimental Materials and Reagents

The reagents included* Spirulina *powder (20150116, Yunnan Green A Biological Engineering Co., Ltd.), papain (20150225, Nanning Pangbo Biological Engineering Co., Ltd.), Dulbecco's modified Eagle's medium (DMEM; Gibco Company), trypsin solution (Gibco Company), and Australian fetal bovine serum (Corning Corporation).

## 3. Screening and Optimization of PSP Extraction Methods

### 3.1. Hot-Water Extraction Method [6, 19, 22]

*Spirulina* powder (40 g) was added to 1.6 kg of water. The mixture was stirred vigorously in a water bath (80°C) for 8 h, followed by centrifugation (4300 rpm, 20 min). The supernatant was concentrated to 1/5 of the original volume. Subsequently, five times the volume of 95% ethanol was added to the concentrated solution. The ethanol mixture was placed in a freezer overnight, followed by centrifuging (4300 rpm) for 10 min. The precipitate was washed by acetone, suction-filtered, and then dried. The polysaccharide extraction rate (polysaccharide mass/*Spirulina* powder dry weight × 100%) and protein content (protein mass/crude polysaccharide mass × 100%) were determined.

### 3.2. Lye Extraction Method [23]

*Spirulina *powder (40 g) was added to 1.6 kg of water, and the pH was adjusted to 10.0 by adding 1 mol/L NaOH. The succeeding experimental steps were the same as those in Section 3.1.

### 3.3. Ultrasound-Assisted Extraction Method [20]

*Spirulina* powder (40 g) was added to 1.6 kg of water, and the mixture was subjected to ultrasonication for 50 min (50°C, 320 W). The remaining experimental steps were the same as those in Section 3.1.

### 3.4. Freeze-Thaw Method

*Spirulina* powder (40 g) was added to 1.6 kg of water. Then, the mixture was frozen at 4°C for 1 h and hot-melted at 30°C for 1 h. These steps were repeated three times, and the subsequent experimental steps were the same as those in Section 3.1.

### 3.5. Single-Factor Inspection of Lye Extraction Method and Response Surface Methodology [23]

The effects of the solid-liquid ratio (1 : 10, 1 : 20, 1 : 30, 1 : 40, and 1 : 50), pH (7.0, 8.0, 9.0, 10.0, and 11.0), temperature (50°C, 60°C, 70°C, 80°C, and 90°C), and time (6, 7, 8, 9, and 10 h) on the extraction rate were studied. Then, several levels with a considerable effect on the polysaccharide extraction rate were selected in accordance with the single-factor test. Response surface design was performed, with the polysaccharide extraction rate and protein content as the response values (Table 1). Extract the PSP according to the experimental schemes provided by Design-Expert® Version 8.0 software. Then get the optimal prediction prescription by analyzing the results in Design-Expert. Subsequently, the optimal extraction prescription was verified. All experiments were repeated three times.

### 3.6. Verification of the Optimal Extraction Process

The optimal extraction process (solid-liquid ratio = 1 : 50, pH = 10.25, temperature = 90°C, and time = 10 h) based on the aforementioned response surface design was repeated three times and then the extraction rate was determined.

### 3.7. Deproteinization and Decolorization

The crude polysaccharide was dissolved in distilled water, adjusted to pH 7.0 by hydrochloric acid, and then incubated at 50°C for 2.5 h after adding 3% papain. Subsequently, the mixture was boiled to inactivate the enzyme and then kept at 4°C overnight after 5% trichloroacetic acid (TCA) was added. The mixture was centrifuged to get the supernatant, which precipitated overnight again with 5% TCA. The fully deproteinized polysaccharide solution maintained at 55°C was adjusted to pH 8.0 by concentrated ammonia, following addition of H_2_O_2_ solution (30%) which kept a 5% final concentration for 2 h. After that, five times the volume of 95% ethanol was added and stored at 4°C overnight to precipitate. The precipitate was dissolved in distilled water and the precipitation process was repeated with 95% ethanol three times. The final precipitate was washed consecutively with anhydrous ethanol, acetone, and ether and then dried. And the protein content and polysaccharide extraction rate were calculated.

### 3.8. Determination of Polysaccharide Components

Crude polysaccharides (200 mg) after deproteinization and depigmentation were dissolved by 2 ml of 72% (w/w) sulfuric acid, followed by water bath at 30°C for 1 h. Then the solution was diluted by ultrapure water to a final sulfuric acid at 4% (w/w), subjected to hydrolysis at 121°C for 1 h, and filtered by 0.45 um membrane. The filtrate was neutralized with BaCO_3_ and then left at 4°C for 24 hours and was then filtered again to remove the precipitate; then, the final hydrolyzate was obtained.

High Performance Liquid Chromatography (Agilent 1260, RI) conditions are as follows: column ZORBAX NH_2_ (4.6 × 250 mm, 5 um), mobile phase (75% acetonitrile in water), column temperature (35°C), injection volume (10 ul), and flow rate (0.8 ml/min).

## 4. Effect of PSP on HaCaT Cell Wound Healing

HaCaT cells were cultured in RPMI-1640 medium supplemented with 10% FBS and 1% penicillin-streptomycin under a humidified, 5% CO_2_ atmosphere at 37°C. Cells were used for experiments at 80% confluency. Cells were incubated at a cell density of 5 × 10^5^ cells/well overnight in six-well plates (Corning, NY, USA), which were marked with equidistant horizontal lines through marker strokes. When the cells were 100% fused, the medium was then removed and longitudinal scratches were made on the cell monolayer using the tip of a 10 *μ*L pipette according to the mark line on the back of the board. This procedure produced the cell wound model. After scratching, the cells were washed three times with PBS, and the exfoliated cells were washed down. These cells were randomly divided into three groups: the normal group (only culture medium was added), the experimental group (the best concentration of proliferation in the cell proliferation experiment), and the positive control group (20 ng/mL growth factor bFGF). 2 mL of fresh medium in different treatment was added at the indicated time, and the distance between scratches was observed under an inverted microscope.

## 5. Results

### 5.1. Selection of Different PSP Extraction Methods

Aliquot dry* Spirulina* powder was subjected to the hot-water extraction method (Method 1), alkaline extraction (Method 2), ultrasonic-assisted extraction (Method 3), or freeze-thaw method (Method 4). A light blue-green powder was eventually obtained. The results are presented in Table 2.

### 5.2. Single-Factor Test

**Figure 1:**
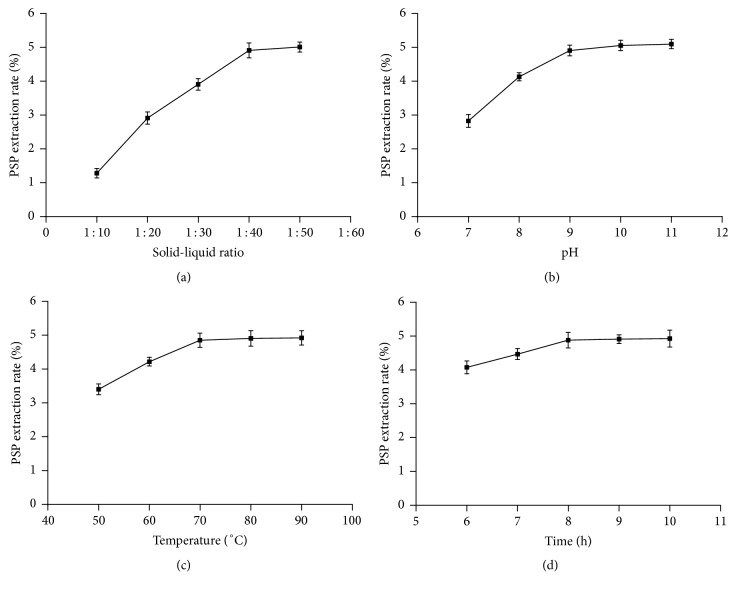
Effects of different ratios of liquid to material (a), pH (b), temperature (c), and time (d) on the extraction rate of PSP.

**Figure 2:**
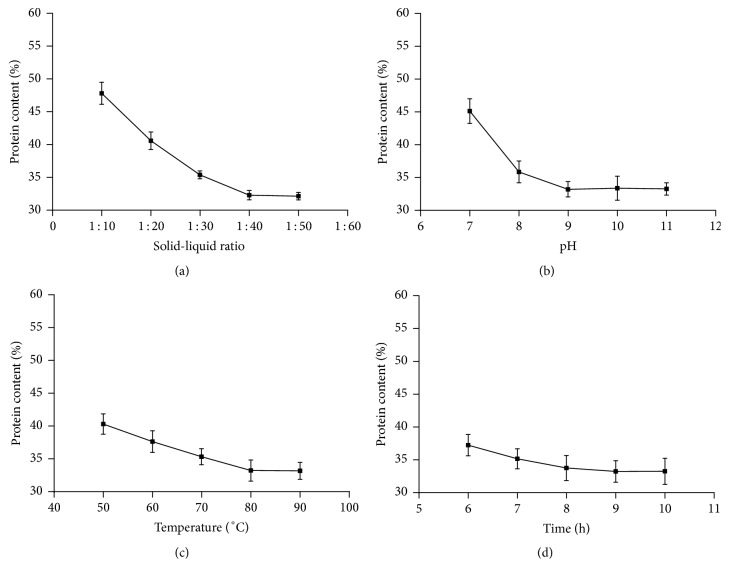
Effect of different ratios of liquid to material (a), pH (b), temperature (c), and time (d) on protein content.

The material-to-liquid ratio, pH, temperature, and time were considered as the abscissas, while the polysaccharide extraction rate and protein content were considered as the ordinates. Trend lines were drawn based on the different conditions. The results are presented in Figures 1 and 2. As shown, the effects of different factors on the polysaccharide extraction rate follow the order of material-to-liquid ratio > pH > water bath temperature > extraction time. Meanwhile, the effects of different factors on protein content follow the order of pH > material-to-liquid ratio > water bath temperature > extraction time. In addition, low polysaccharide extraction rate and low protein content were obtained at the material-to-liquid ratios of 1 : 30, 1 : 40, and 1 : 50; pH values of 8.0, 9.0, and 10.0; temperatures of 70°C, 80°C, and 90°C; and extraction times of 8, 9, and 10 h. Therefore, these levels were selected for response surface optimization while designing the response surface scheme.

### 5.3. Optimal Process and Verification of Response Surface Design

Crude polysaccharide extraction was performed according to the 29 groups from the experimental program suggested by the Design-Expert 8.0.6 software. Then, polysaccharide extraction rates and protein contents were determined. The material-to-liquid ratio, pH, temperature, and time were considered as independent variables, whereas the polysaccharide extraction rate and protein content were considered as dependent variables. Multiple linear regression and binary polynomial regression were conducted, and goodness of fit (*r*) and confidence (*p*) were used as standard models for judging the result. The results show that the binary polynomial model is better than multiple linear regression, and the model equations are as follows:

(1) Y1=4.91+0.29X1+0.052X2+0.067X3+0.042X4+0.018X1X2−0.23X12−0.012X22−0.02X32+0.073X42,where  r=0.9915.

*X*_1_, *X*_2_, *X*_3_, and *X*_1_^2^ (*p* < 0.01) in these coefficients were highly significant, whereas the others were insignificant.

(2) Y2=33.14−1.02X1−1.26X2−0.85X3−0.41X4+0.42X3X4+0.64X12+0.49X32,where  r=0.9309.

*X*_1_, *X*_2_, and *X*_3_ (*p* < 0.01) in these coefficients were highly significant, whereas the others were insignificant.

**Figure 3:**
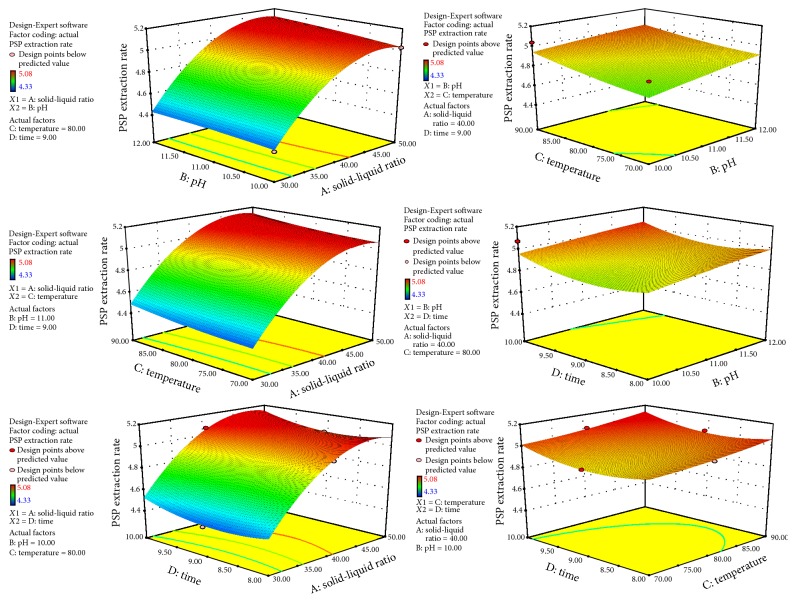
3D response surface scheme of factors and the response values.

To observe the interaction between various factors intuitively, we used the Response-Expert® 8.0.6 software continuously for response surface analysis, and a 3D response surface map was produced on the basis of the results of the binary polynomial model fitting (Figure 3). The optimal extraction process predicted by the software involved the following conditions: material-to-liquid ratio = 1 : 50, pH = 10.25, water bath temperature = 89.24°C, extraction time = 9.99 h, predicted PSP extraction rate = 5.21%, and protein content = 30.66%. To facilitate the operation, we chose temperature to be 90°C and the extraction time to be 10 h. The other conditions remained unchanged. Three batches of samples were prepared in parallel, and the optimal prescription was verified by measuring the PSP extraction rate and protein content. The results are shown in Table 3. The relative deviations of the PSP extraction rate and protein content were less than 2%. This result revealed that the model can effectively reflect the relationship between the indicators and factors.

### 5.4. Deproteinization and Decolorization Treatment

The extracted PSP was deproteinized by the enzymatic TCA method. The obtained polysaccharide content was 65.71 ± 0.93% and the protein content was 10.15 ± 0.14%. Then, the decolorization was continued, and the polysaccharide and protein contents were obtained as 71.65 ± 1.12% and 8.54 ± 0.12%, respectively.

### 5.5. Polysaccharide Components

The results of the monosaccharide composition of PSP analyzed by High Performance Liquid Chromatography are shown in Table 4.

### 5.6. Effect of PSP on HaCaT Cell Wound Healing

**Figure 4:**
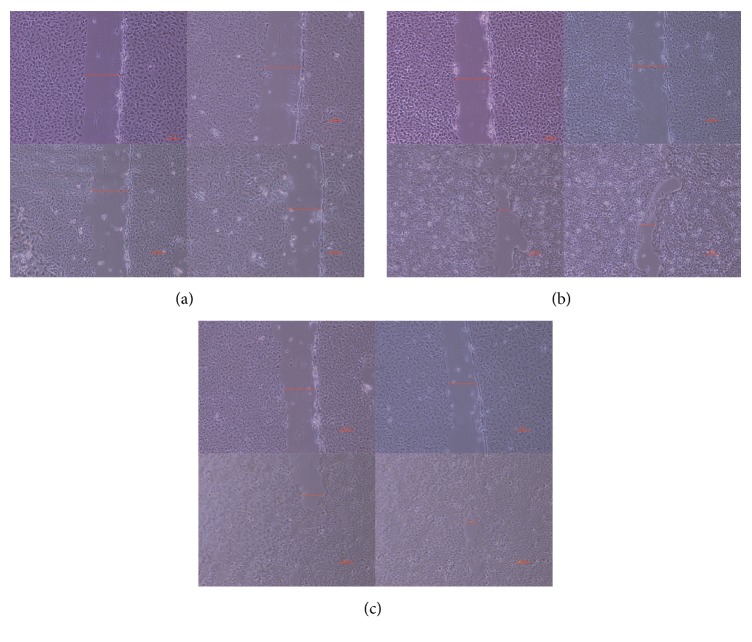
Cell scratch test of PSP on HaCaT.

The scratch width of the PSP experimental group (b) and the positive control group with bFGF (c) presented a clear narrowing trend, whereas the scratch width of the normal control group (a) was not significant (Figure 4). Thus, we conclude that the PSP participated in promoting HaCaT cell migration and fusion, which was similarly to the growth factor bFGF. Thus, PSP exhibited a good effect on wound healing.

## 6. Discussion and Conclusion

In this study, polysaccharide and protein analysis methods were first established. Then, the optimal polysaccharide extraction method was selected among hot-water extraction, alkali extraction, ultrasound-assisted extraction, and the freeze-thaw method. Hot-water extraction does not require special equipment but has lower efficiency than others. Alkali extraction and ultrasound-assisted extraction were developed based on hot-water extraction. Some plant polysaccharides, particularly acidic plant polysaccharides, exhibit high solubility in alkaline solutions. Alkaline solutions are also beneficial for breaking cell walls and releasing polysaccharides to obtain increased extraction rates. However, the alkalinity and temperature must be controlled to prevent polysaccharide hydrolysis. We should pay close attention to alkalinity and temperature control because inappropriate operation may cause polysaccharide hydrolysis. Ultrasound-assisted extraction mainly uses ultrasound to break cells, while the freeze-thaw method involves flash freezing at a low temperature and then gradual melting. The two operations were repeated multiple times to break the cell wall. The principle is as follows: the hydrophobic bond structure is broken after being frozen. Meanwhile, intracellular ice crystals are produced which disrupt the cell wall mechanically. The experimental results showed that the alkali extraction method has a higher efficiency than those of other methods. After univariate analysis, the three most influential factors were used for the analysis of response surface experimental design. In the present experiment, the optimal extraction process was predicted to involve the following conditions: material-to-liquid ratio = 1 : 50, pH = 10.25, water bath temperature = 89.24°C, extraction time = 9.99 h, predicted PSP extraction rate = 5.21%, and protein content = 30.66%. Then, the extraction process was verified. The crude polysaccharide in blue-green color was obtained, but it contained high protein. In order to remove the protein, we performed Sevage, enzymatic, enzymatic-Sevage, TCA, and enzymatic hydrolysis-TCA methods and compared the deproteinization effects. The enzymatic hydrolysis-TCA method exhibited a better deproteinization effect than others and yielded a protein content of 10.15%. The Sevage method was a protein removal method with a long history. The protein precipitated by denaturation was caused by chloroform,* n*-butanol, and other organic reagents. However, this method which requires repeated processes is unable to denature the proteins tightly bound to polysaccharides and may cause organic solvent residue. The enzymatic protein removal can overcome this problem. However, enzymatic reaction requires increased stringency of the control of conditions. Moreover, the enzyme itself is a kind of protein and may contaminate the sample. In contrast, the TCA method of protein precipitation is more thorough and can remove small amounts of pigment. However, this method strictly requires a certain pH to prevent potential polysaccharide hydrolysis. Considering the advantages and disadvantages of each protein removal method, several techniques are generally combined to improve the protein removal efficiency. When the protein portion was removed, the polysaccharide became light green.* Spirulina *contains chlorophyll and other pigments. The pigments need to be removed, and oxidative decolorization is the commonly used method. H_2_O_2_ is rapidly decomposed when heated, which generates nascent oxygen and destroys the colored material. The method exerts a decolorizing effect on polysaccharides. Finally, a pale-yellow PSP powder with a polysaccharide content of 71.65% and a protein content of 8.54 was obtained.

Scratch experiments were performed to compare the skin-damage-repairing effects between the PSP and the growth factor. The effects were found to be almost comparable, suggesting that the PSP exerts good effect on skin repair.

## Figures and Tables

**Table 1:** Factors and levels for RSD.

Factors	Levels		
	−1	0	1
Solid-liquid ratio (*X*_1_)	30	40	50
pH (*X*_2_)	8	9	10
Temperature (*X*_3_)	70	80	90
Time (*X*_4_)	8	9	10

**Table 2:** Extraction rate, polysaccharide content, and protein content of crude PSP obtained by different extraction methods (*n* = 3).

Method	Extraction rate (%)	Polysaccharide content (%)	Protein content (%)
1	3.52 ± 0.04	9.53 ± 0.10	41.91 ± 0.77
2	4.37 ± 0.05	10.8 ± 0.13	34.8 ± 0.53
3	3.88 ± 0.05	8.97 ± 0.08	40.17 ± 0.44
4	3.97 ± 0.07	10.16 ± 0.16	47.7 ± 0.78

**Table 3:** Verification test of the optimized extraction process by RSD (*n* = 3).

Index	Predicted value	Actual value	RSD (%)
PSP extraction rate (%)	5.21	5.19 ± 0.02	0.38
Protein content (%)	30.66	30.96 ± 0.09	0.29

**Table 4:** Monosaccharide composition of crude polysaccharide (%).

Glucose	Rhamnose	Xylose	Galactose	Arabinose	Unknown 1	Unknown 2
21.3 ± 1.4	43.6 ± 2.7	2.4 ± 0.6	1.3 ± 0.2	1.1 ± 0.1	1.5 ± 0.3	0.7 ± 0.4
